# Fluorine-19 MRI signal phenotype in solid tumor following in situ perfluorocarbon cell labeling

**DOI:** 10.1038/s41598-026-54741-4

**Published:** 2026-06-03

**Authors:** Eric T. Ahrens, Keith Tang, Giuliana P. Mognol, Hui Chen, Benjamin I. Leach, Andrew Davis, Judith Varner

**Affiliations:** 1 Department of Radiology, University of California, San Diego, 9500 Gilman Dr. #0695, La Jolla, CA 92093-0695 USA; 2 Department of Pathology, University of California, San Diego, La Jolla, CA USA; 3 GondolaBio, Palo Alto, CA USA

**Keywords:** MRI, Fluorine-19, Perfluorocarbon, Nanoemulsion, Macrophage, Inflammation, Biomarkers, Cancer, Immunology, Oncology

## Abstract

Cell tracking with perfluorocarbon (PFC) nanoemulsion (NE) and ¹⁹F MRI has been used in numerous inflammation models, including tumors. After intravenous injection, phagocytic immune cells endocytose NE droplets, producing background-free ¹⁹F images where signal foci quantify inflammation. Histology confirms tracer uptake mainly in myeloid-lineage cells. Myeloid cells - granulocytes, monocytes, and macrophages - play major roles in cancer. Tumor-associated macrophages (TAMs), which can comprise up to 60% of tumor mass, promote angiogenesis, metastasis, and immunosuppression. High TAM burden correlates with poor prognosis in several cancers and reduced responsiveness to immunotherapies. In this study, we characterize ¹⁹F MRI signals, following in situ NE labeling, in a syngeneic breast cancer model. Our goal is to validate the cell phenotype responsible for the ^19^F signals observed in the tumor microenvironment. We quantified ¹⁹F signal contributions from myeloid subsets using flow cytometry and ¹⁹F NMR and mapped intratumoral distributions via cryo-fluorescence tomography. These results demonstrate selective NE uptake by myeloid cells and support ¹⁹F MRI as a noninvasive biomarker for TAM burden in cancer.

## Introduction

In situ immune cell labeling with colloidal perfluorocarbon (PFC) nanoemulsion (NE) tracers, combined with ^19^F MRI detection, has been effectively used in numerous preclinical inflammation models^1–13^, including in solid tumors (e.g., Refs.^14–18^. Following intravenous injection, phagocytic immune cells rapidly endocytose the NE droplets, thereby labeling cells of the reticuloendothelial system (RES)^19^. Accumulation of PFC-labeled phagocytes at inflammatory sites yields pure ^19^F MRI hot-spot images with no background signal. These ^19^F MRI foci can be quantified to determine the absolute number of fluorine atoms^2,5,9^, a metric proportional to inflammation severity.

Myeloid cells, including granulocytes, monocytes, and macrophages, can play key roles in cancer progression and therapeutic response. These cells can adopt pro- or anti-tumor states, depending upon cues within the tumor microenvironment. Granulocytes can promote tumor growth and metastasis through the production of cytokines, metalloproteinases, and neutrophil extracellular traps, which are net-like DNA-protein complexes that entrap bacteria and promote metastasis^20^. Monocytes can contribute to tumor progression by serving as precursors to macrophages^21^. Tumor-associated macrophages (TAMs) can comprise 20–60% of the tumor mass and serve several pro-tumoral functions, including expression of cytokines that inhibit T-cell recruitment and survival, or promote angiogenesis and metastasis^22^. Moreover, TAMs release matrix metalloproteinases that suppress adaptive immune responses^23^, and secrete chemokines that attract non-cytotoxic T-cell subsets, further facilitating tumor progression and metastasis^24^.

Numerous studies suggest that TAM burden (i.e., density) within tumors may be predictive of disease course and therapeutic response. Increased macrophage infiltration is often associated with advanced-stage disease and poorer overall survival in breast^25^, pancreatic^26^, and bladder cancers^27^. In contrast, high macrophage density in colorectal cancer has been correlated with favorable outcomes^28^. However, other studies have shown that TAMs can promote immune suppression and metastasis in later stage colorectal carcinomas^29^. TAM burden may also serve as a prognostic biomarker for treatment response. For instance, in Hodgkin’s lymphoma, a high burden of CD68 + macrophages (a pan-macrophage marker) is associated with primary treatment failure and poor survival^30^. Similarly, in pancreatic cancer, TAM burden is a key prognostic indicator for post-surgical chemotherapy response^31^.

Immune checkpoint inhibitors (e.g., anti-PD-1 and anti-CTLA-4 antibodies) have revolutionized cancer therapy over the past decade^32,33^. However, response rates remain low; for example, PD-1 blockade benefits fewer than 20% of triple-negative breast cancer patients^34^. A high TAM burden in the tumor microenvironment (TME) is associated with poor prognosis and diminished responses to such therapies^22,35^. TAMs promote immunosuppression and T-cell exclusion, both linked to low responsiveness to checkpoint inhibitors. Given their abundance and role in tumor progression, a quantitative TAM imaging biomarker could substantially enhance our ability to diagnose, stratify, and monitor cancer treatment efficacy with current and future immunotherapies.

In this study, our goal is to validate the ^19^F MRI signal phenotype in the TME following in situ cell labeling with a PFC NE tracer. Experiments were performed in a syngeneic breast cancer rodent model, where the distribution and phenotype of labeled cells within the TME were characterized by flow cytometry. We estimated the relative ^19^F MRI signal contributions from different myeloid cell subsets by combining flow cytometry of dissociated tumor tissues with quantitative ^19^F NMR analyses of immuno-cell lines. Furthermore, we visualized the three-dimensional (3D) intratumoral biodistribution of PFC-labeled phagocytes throughout the entire tumor mass using cryo-fluorescent tomography (CFT)^36^. To enable these studies, we employed composite dual-mode NE agents detectable by both ^19^F MRI and fluorescence^37^. Overall, these studies demonstrate the high specificity of NE agents for myeloid cells and provide insight into the biological significance of the observable ^19^F MRI signals in the TME, thereby supporting use as a TAM imaging biomarker.

## Results

### In vivo imaging

**Fig. 1:**
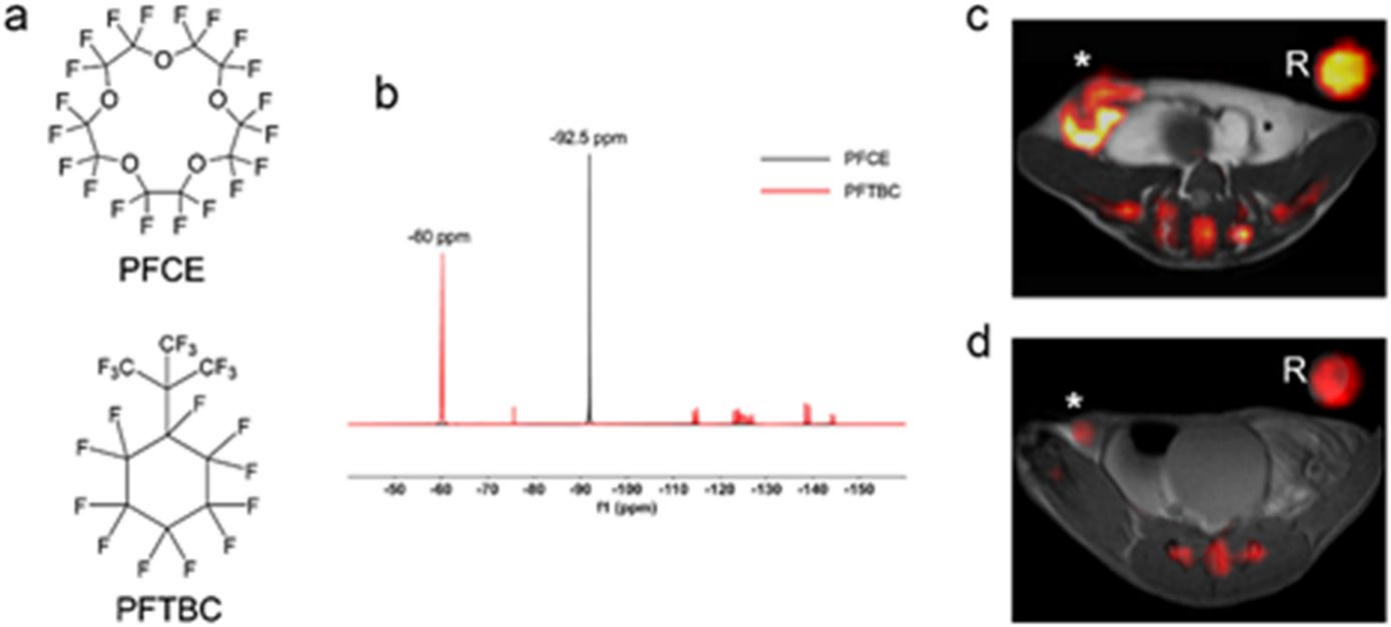
In vivo ^19^F MRI in 4T1 tumor mice. Panel (**a**) displays representative PFC molecules used for NE formulation and ^19^F MRI, including perfluoro-15-crown-5 ether (PFCE) and perfluoro-tert-butyl-cyclohexane (PFTBC), where (**b**) is an overlay of the ^19^F spectra for these molecules. In (**c**), we show ^1^H (grayscale) and ^19^F (pseudo-color) MRI of mice bearing 4T1 tumor that were injected with PFCE or PFTBC NE. Signal hot-spots are observed in the tumor (asterisks), and ^19^F signals are also observed in bone marrow. “R” is external capillary reference tube. Data were acquired 24 h after NE injection in mice with established tumors.

Following intravenous administration, PFC NE is effective in labeling cells in the TME that are detectable using ^19^F MRI. Figure 1 displays representative MRI results in an orthotopic breast cancer 4T1 mouse model 24 h after tracer administration. In the same model, two different tracer compositions are displayed (Fig. 1a), including PFTBC (perfluoro tert-butylcyclohexane) and PFCE (perfluoro-15-crown-5 ether). PFTBC and PFCE are example NE tracers often used in ^19^F MRI studies. Of these, PFCE has the better ^19^F MRI sensitivity due to 20 equivalent F-spins and often used in preclinical (animal) studies. However, PFCE is not well suited for human use due to its long retention time in the body > 100 days, especially for the high doses used for in situ myeloid cell labeling methods. In contrast, PFTBC clears the body faster (~ 10 days), and thus is better suited for potential clinical applications.^38^ However, PFTBC has a more complex ^19^F spectrum and thus lower intrinsic sensitivity as only a portion of the ^19^F molecular sites are used for image generation (Fig. 1b). The surfactant and manufacturing process are the same for both molecules, and formulated tracers have a similar particle size. Thus, the PFC choice depends on whether the study is animal-only or human translational. As expected, both molecules display similar MRI readouts and have comparable biodistribution. Figure 1c, d displays ^19^F/^1^H image transaxial slices through the lower abdomen centered over the mammary fat pad tumor. All images show ^19^F signal hot-spots localized within the tumor (asterisks), as well as in lymph nodes and bone marrow, as expected for RES uptake. The signal intensity of the PFTBC injected mouse is lower overall due to the data acquisition form only a subset of spins at the − 60 ppm peak (Fig. 1b) using a narrow bandwidth pulse to avoid chemical shift artifacts from neighboring peaks. The data acquired for the − 60 ppm PFTBC peak represents 45% (9/20) of the total ^19^F spins. In contrast, PFCE has a single ^19^F spin resonance (-92.5 ppm, Fig. 1b), and thus has higher intrinsic sensitivity due to signal acquisition of all 20 equivalent spins. Quantification of the absolute number of apparent ^19^F spins in the three-dimensional tumor volume (Fig. 1c, d), aided by the reference capillary alongside the torso (“R”), yield an average (*N* = 3) of 1.0 × 10^21^ and 2.3 × 10^21^ fluorine-19 spins for PFTBC and PFCE, respectively, with a ratio of 43%, which is close to the expected theoretical sensitivity ratio for the two molecules (45%).

### Visualization of 3D distribution of labeled leukocytes in tumor

**Fig. 2:**
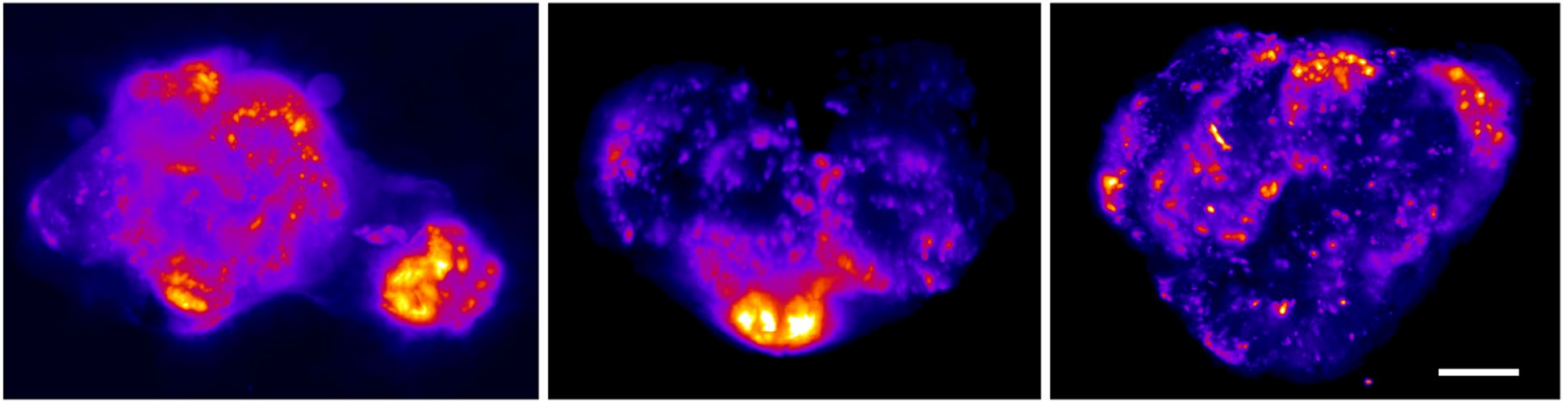
Visualization of 3D distribution of DiI-PFC+ cells in whole tumor. We display 3D histological data acquired in three excised tumors using CFT showing the spatial distribution of labeled cells throughout the entire tumor. The 3D data is rendered in pseudo-color as a maximum image projection. Image data were acquired at 18.3 × 50 × 50 μm resolution. Scale bar is 2 mm.

In excised tumors, we used cryo-fluorescence tomography (CFT) to visualize the NE distribution and labeled cell distribution in tumor mass (Fig. 2). CFT is a three-dimensional histology technique^39^ where the entire animal or intact organ-tissue is mounted in frozen cryo-embedding compound *en bloc* and sectioned using a robotic macrotome. White-light and fluorescence images are acquired of the block face after each cut. These data are reconstructed into high-resolution 3D images. For these studies we used a dual-mode, fluorescently-tagged NE composition^40^. Figure 2 displays maximum intensity projection (MIP) images of three different 4T1 tumors. The NE tracer displays a highly punctate heterogenous NE distribution, particularly in the tumor marginal zone.

### ^19^F cell-signal phenotype

**Fig. 3:**
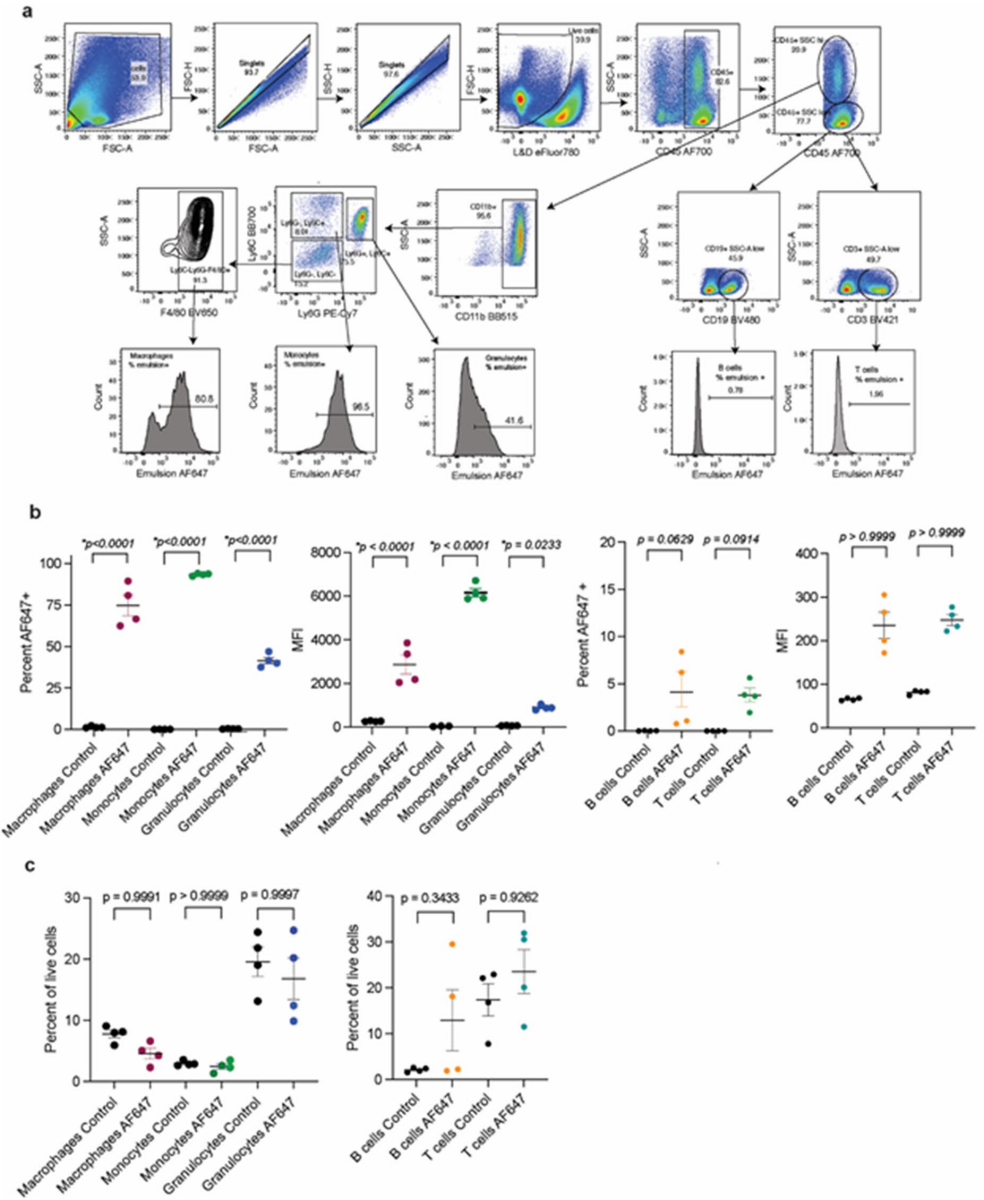
Phenotypic profiling of PFC labeled immune cells in TME. (**a**) Representative flow cytometry gating strategy of immune populations isolated from 4T1 tumors 48 h after tail injection with control or AF647-labeled PFCE NE. (**b**) Quantification of percent AF647 + cells and mean fluorescent intensity (MFI) of each immune subpopulation (myeloid lineages left and lymphoid lineages right, *n* = 4 per group). (**c**) Quantification of each immune subpopulation in tumors as a percentage of total live cells. (*n* = 4 per group). All data is expressed as mean +/-− SEM. Statistical significance was determined by one-way ANOVA with Tukey’s post-hoc test for multiple group analysis, where *p < 0.05 indicates significance.

In the 4T1 breast cancer model, tumors were analyzed using flow cytometry to assess the labeling of immune cell phenotypes following injection with dual-mode PFC NE harboring an Alexa Fluor (AF647) fluorophore. Tumors were excised, dissociated into single-cell suspensions using standard methods, and were immunostained to detect immune cell (CD45), myeloid cell (CD11b, Ly6C, Ly6G, F4/80), T cell (CD3) and B cell (CD19) using fluorescently labeled antibodies. Percentages of each cell type were assessed by flow cytometry in four replicate tumor samples with 4.7 × 10^5^ to 7.5 × 10^5^ cells analyzed per condition. Post-processing gating identified and quantified live cells, CD45 + immune cells, CD19 + B cells, CD3 + T cells and specific subpopulations of myeloid cells, including CD11b+ Ly6C+ Ly6G- monocytes, CD11b+ Ly6C+ Ly6G+ granulocytes and CD11b+ Ly6C- Ly6G- F4/80 + macrophages (Fig. 3a). The percentage of each immune cell population taking up AF647 + NE, as well as the subsequent mean fluorescence intensity of each population, are shown in Fig. 3b. These results show that myeloid cells, but not T cells or B cells, are efficiently labeled with NE (Fig. 3a-b). Whereas a small amount of NE emulsion is taken up by about 40% of granulocytes, nearly all monocytes (96%) and macrophages (81%) are NE positive and exhibit high levels of fluorescence (Fig. 3b). Although granulocytes were the most abundant myeloid cells in 4T1 tumors (75% of myeloid cells, Fig. 3c), they exhibited the lowest NE uptake and lowest mean fluorescence of myeloid cell populations (Fig. 3b). Together, macrophages and monocytes constitute 25% of myeloid cells in tumors yet uptake most of the emulsion (Fig. 3b-c). Overall, the tumor tissue contains significantly labeled AF647 + bone marrow-derived monocytes and macrophages; Ly6G+ neutrophils are more-weakly positive for NE but are more abundant in 4T1 tumors. Negative control mice, without AF647-PFC NE injection, displayed no signal in AF647 channel. In the T/B cell panel, cell uptake was detectable, but low (< 1% AF647+, Fig. 3b).

### Quantitative update of PFC in immune cells via ^19^F NMR

**Fig. 4:**
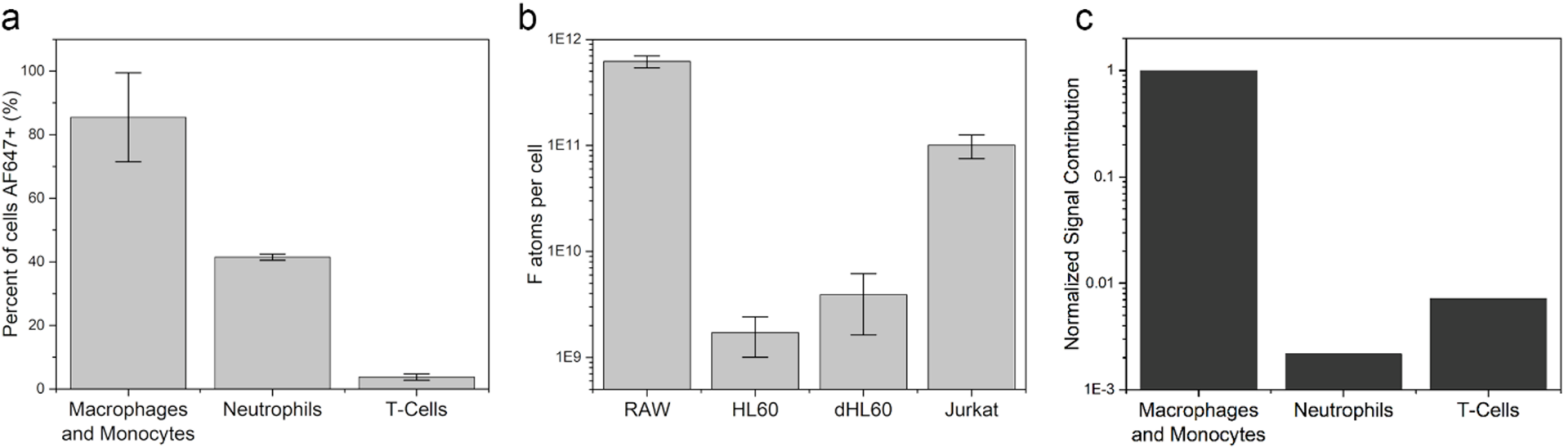
Modeling of relative ^19^F signal contribution of macrophages/monocytes versus neutrophils and T cells in TME. Panel (**a**) displays the percentage of a given cell type labeled with PFC-AF647 NE observed in flow cytometry (Fig. 3) in the disassociated tumor tissue samples. In (**b**), we show the absolute mean ^19^F/cell uptake in vitro following co-incubation of PFCE NE with different cell lines that model macrophage/monocyte (RAW), neutrophil (HL60 & DHL60) and T cell (Jurkat) phenotypes. Measurements were made using quantitative ^19^F NMR spectroscopy of cell pellets following a 12 h co-incubation followed by wash (*N* = 3 replicates). The (log scale) bars in (**c**) represent the product of (**a**) and (**b**) data, providing an estimate of the relative ^19^F signal contributions of the major cell types present in the TME. For neutrophils, the average uptake value for HL60 and DHL60 lines is used. Overall, monocytes/macrophages represent the dominate signal contributor by two orders of magnitude.

The contribution of the different immune cells present in the TME to the observable ^19^F MRI signal in vivo is a function of the number of cells of a given phenotype present in the tumor, the amount (mass) of tracer that a given immune cell can intrinsically take up, and the limit of detection (LOD) of the MRI technique^41^. Flow cytometry analysis of single cell tumor suspensions (Figs. 3 and 4a) provides an assay of the frequency of finding a particular labeled cell phenotype in tumor. However, different immune cell types have widely varying cytoplasmic volumes and endocytic abilities, which limit the amount of tracer that a given cell type can take up, and thus its intrinsic detectability within a voxel. Using in vitro labeling studies, where PFC NE is co-incubated with cell cultures for a 12 h period, one can quantitively measure the mean ^19^F/cell in different cell types^40,42^; these data can be used to model their potential to contribute to the observable signal. For example, Fig. 4b displays in vitro PFC NE labeling studies in different cell lines including macrophage/monocyte (RAW), T cells (Jurkat), and neutrophils (HL60 & DHL60). Macrophages dominate uptake, with T cells and neutrophils taking up ~ 10^1^-10^2^-fold less. Figure 4c displays a modeling estimation of the relative signal contribution of different cell types observed in ^19^F tumor hot-spot scans, calculated by multiplying flow cytometry frequency data (Fig. 4a) and in vitro ^19^F cell uptake data (Fig. 4b). The modeling shows that macrophages/monocytes are at least 100-times more detectable on a per-cell basis compared to neutrophils or T cells. Importantly, the LOD of the MRI technique set the lower limit of cell detectability, and generally ^19^F MRI hotspots are in the low signal-to-noise regime (< 10). Thus, cell populations with low signal contributions (Fig. 4c) fall below the LOD and do not appreciable contribute to the measured ^19^F signal in vivo.

## Discussion

Assaying TAMs non-invasively may be valuable for personalized cancer care, treatment planning, and monitoring. An image-based biomarker for TAMs using non-targeted PFC tracers and ^19^F MRI provides a quantitative readout of TAM density in solid tumors. The net signal detected in a voxel represents a superposition of labeled cells comprised of a repertoire of cell types. Overall, myeloid cells in the TME, particularly macrophage/monocyte cell populations, are the dominant signal contribution to ^19^F MRI signals in a solid tumor model system. The syngeneic 4T1 breast tumor model in these studies is a TAM-rich model that was used to validate NE ¹⁹F MRI as a noninvasive biomarker for TAM burden in cancer. A limitation of these studies is that the TME and macrophage content of solid tumors can vary significantly by tumor type and stage. Future studies that investigate additional tumor models characterized by high and low myeloid cell compositions and diverse distributions will enhance the general applicability of these results.

In vivo fluorine-19 MRI using F-based tracer media has shown utility and versatility for a range of pre- and clinical biomedical uses, particularly immune and stem cell detection, respiratory airway imaging, as well as biosensing^43^. Overall, use of PFC tracers has several advantages^40,44^ over ^1^H-based contrast agent approaches^45^ because it yields robust quantification, an absence of false positive signals at lesion sites, and does not require a pre-contrast scan. PFC molecules are the ^19^F signal generators in the majority of cell tracking applications due to their high fluorine density and bioinertness^46^. The ^19^F nucleus intrinsically sensitive, as it has a similar gyromagnetic ratio to proton (^1^H) differing by only ~ 6%, spin ½, and 100% natural abundance.

The notion of using PFC NE to visualize solid tumors dates back to the 1980s^13,47^, and these studies were among the earliest implementations of ^19^F MRI. What has evolved since then is the biological interpretation of the apparent ^19^F image hotspots observed in tumors. The enhanced permeability and retention (EPR) effect is often cited as the source of ^19^F tumor signal. However, we consider it unlikely that EPR can be visualized directly, as the ^19^F concentration in tumor tissue during the early phase of blood extravasation is far below the in vivo LOD. Tumor signals become visible only after PFC droplets have cleared the bloodstream and subsequently sequestered and concentrated within lysosomal vesicles^40^ of phagocytic myeloid cells. Small particulates in the body, such as the ~ 180 nm NE droplets, do not remain “free” for long, of order only minutes to hours, because they are rapidly endocytosed as part of innate immune surveillance against potential “pathogens”. For the nanoemulsions used herein, the blood half-life is ~ 10 hours^4,48^. Thus, by the 24 h ^19^F MRI timepoint, the bulk of the agent has cleared the blood stream. Longitudinal ¹⁹F signal stability at lesions has previously been demonstrated^1,2^, supporting the option for imaging time windows extending over multiple days.

^19^F NE imaging of inflammation detects the net signal from all labeled cells within a voxel; however, not all labeled cells contribute equally to the observable signal. Empirically, both cytoplasmic volume and intrinsic phagocytic activity are major determinants of the PFC mass taken up by a given cell. In vitro studies of PFC NE labeling show that macrophages can accumulate a large payload, on the order of 10^12^ ^19^F atoms per cell. In contrast, T cells exhibit ~ 10-fold lower uptake (Fig. 4b) due to their intrinsically low phagocytic activity and small cytoplasmic volume, making their contribution to the ^19^F signal negligible. Neutrophils, although phagocytic, are physically smaller than macrophages, with roughly a 4-fold lower volume, and therefore have less intrinsic capacity for NE uptake (Fig. 4b); as a result, their per-cell ^19^F contribution is also substantially lower than that of macrophages. We note that the absolute in situ ^19^F/cell uptake is unknown and is likely lower than the values obtained in vitro. However, the relative contributions of the different cell types to the overall ^19^F signal (Fig. 4c) are presumably preserved.

A large body of supporting data in other types of inflammatory diseases show the specificity of PFC NE macrophage labeling at lesion hot-spots. For example, in mouse models of intestinal inflammation which mimics aspects of inflammatory bowel disease, we have shown exclusive colocalization of fluorescently-conjugated PFC NE and F4/80 + macrophages in immunohistochemistry, as well as correlation between inflammation in the colon wall determined by histopathology and total ^19^F signal in the same specimens^2,49^. Additionally, quantitative PCR analysis of CD68 mRNA showed that macrophage burden is directly correlated with ^19^F signal in colon samples^2^. Moreover, clodronate liposome treatment, which causes macrophage apoptosis, ablates the ^19^F signals in the colon of IBD mice^2^. Moreover, a notable body of work has investigated the use of PFC NE for macrophage imaging in the context of cardiovascular disease models^8,9,50^.

Non-targeted PFC NE in situ labeling as herein is ‘pan-macrophage’, analogous to CD68 + staining. PFC NE has no known labeling specificity between pro-inflammatory and reparative phenotypes. The use of ‘pan-macrophage’ markers, such as CD68 + staining in biopsies, are highly clinically relevant and are routinely used to analyze clinical samples to score inflammation^51^. We note that in untreated tumors, TAMs are predominantly (~ 90%) skewed toward a reparative phenotype^52,53^. Non-targeted PFC NE has the advantage that the image interpretation is robust as it reflects the myeloid cell density in regions of interest. In certain respects, the quantitative readout is analogous to the scoring of CD68 + cell density in histopathological tissue assays, except the readout is non-invasive and in vivo.

Potentially, alternative formulations of PFC NE may incorporate targeting moieties displayed on NE droplet surface, such as antibodies and peptides to bias uptake to a more narrowly-focused cell phenotype. Overall, targeted tracers binding to specific cell surface receptors are limited by steric access of the probe to targeted cells, as well as by receptor expression levels, which can evolve over time or by anatomical location. Thus, the ^19^F MRI signal may not be linearly proportional to cell density which may confound data interpretation. Moreover, the targeted surface receptor may be expressed in irrelevant tissues that are of no interest for the study leading to false positive signals.

CFT displays highly heterogenous distribution of signals over tumor volume (Fig. 2). The TAM spatial distribution in solid tumor reflects both their function and the tumor’s microenvironmental gradients (e.g., oxygen, nutrients, cytokines). Generally, immunosuppressive TAMs tend to accumulate in specific regions that have distinct roles in tumorigenesis^54–57^. These regions include: (i) the tumor periphery or invasive front, where they promote tumor invasion and angiogenesis, (ii) the necrotic core, supporting angiogenesis and survival of tumor cells under stress, (iii) perivascular niches, to aid angiogenesis, as well as pro-metastasis tumor cell efflux, (iv), stromal and interstitial regions, and (v) at the border between tumor nests and infiltrating lymphocytes. Overall, the punctate, heterogenous nature of the TAM localization as seen in the animal CFT data (Fig. 2) highlights why the clinical use of needle biopsies for TAM quantification in patients (e.g., using CD68 + staining) is often prone to sampling error and inaccurate quantification. This limitation motivates, in part, the development of a whole-tumor, non-invasive biomarker for TAMs using ^19^F MRI.

The biocompatibility and pharmacokinetics of PFC NEs are well characterized over many decades of research. The high oxygen solubility of PFCs have made them candidates for oxygen-carrying blood substitutes since the 1980s^58,59^. In the clinic, microbubbles containing gaseous PFC are routinely used for contrast-enhanced ultrasound imaging^60^. Perfluorocarbons have simultaneous lipophobic and hydrophobic properties and do not incorporate into cell membranes^61^. There are no known enzymes that degrade PFCs in vivo, and they do not degrade at typical lysosomal pH values^61^. The intact PFC molecule ultimately clears the body via lung exhalation^62^. PFC emulsions have been rigorously tested for biological safety, with no observed adverse effects to viability or function in cells. Prior studies have investigated the impact of PFC labeling of primary immune cells using a variety of sensitive in vitro assays, for example in the context of murine DCs^40^, T cells^63^, stem cells^64,65^, and mesenchymal stem cells (MSCs)^66^. The most detailed in vitro study involved PFC labeled primary human DCs^67^; cells were assayed for viability, maturation phenotype, cytokine production, T cell stimulatory capacity, and chemotaxis, and no difference in these parameters was observed between labeled and unlabeled cells. Thus, our view is that it may be feasibility to use PFC NE tracers in investigational clinical trials to assay TAM burden in solid tumors and metastases.

## Methods

### PFC nanoemulsion

Nanoemulsions were formulated using a similar protocol as previously described^36,48^. In brief, egg yolk phospholipid 103 mg (Sigma Aldrich, St. Louis, MO), 6 mg cholesterol (Avanti Polar Lipids, Alabaster, AL), 10 mg DSPE-PEG-2000 (Nanosoft Polymers, Winston-Salem, NC) were prepared by dissolving in chloroform and drying as a thin film in a glass vial under argon, then further under vacuum overnight. Lipids were resuspended in 4.5 ml water with 122 mg mannitol (Sigma Aldrich, St. Louis, MO) by vortexing for 1 min, followed by sonication (Omni Ruptor 250 W, 30% power, 2 min, Omni International, Kennesaw, GA). 1.2 ml of PFCE (Exfluor Research Corporation, Round Rock, TX) or PFTBC (FluoroMed L.P., Round Rock, TX) was added and the mixture vortexed for 10 s followed by sonication for 2 min. Crude emulsions were passed through a microfluidizer (LV-1, Microfluidics, Westwood, MA) at 20,000 psi five times. Optionally, NE was fluorescently labeled by addition of 5 µl of a 10 µM solution of DiI (1,1’-dioctadecyl-3,3,3’,3’-tetramethylindocarbocyanine perchlorate, Thermo Fisher, Waltham, MA) and immediately mixed by swirling the vial. Alternatively, fluorescent tracer was prepared using “click-ready” NE^68^, prepared similarly to above, except with the addition DSPE-PEG2000-DBCO (BroadPharm, San Diego, CA) in surfactant. NE was then fluorescently labeled by adding 152 µL of a 1 mg/mL AF647-N_3_ stock solution in DMSO (dimethyl sulfoxide) to 4 mL NE and mixed overnight at 4 °C. All NEs were desalted into buffer for intravenous injection with 10 mM Tris pH 7.5 and 2% propylene glycol (Sigma Aldrich, St. Louis, MO) using a Nap-10 column (GE Healthcare, Chicago, IL) and filtered through a 0.22 μm filter into sterile glass vials. Concentrations were determined relative to a TFA reference by ^19^F NMR.

### Murine tumor model

4T1-Luc2 cells (ATCC, CRL-2539-LUC2™) were maintained in RPMI-1640 medium containing 10% fetal bovine serum (FBS) and 8 µg/ml Blasticidin. Five million cells suspended in 50 µl PBS containing 50% matrigel were inoculated to the fourth mammary fat pad of female BALB/c mice (6–8 weeks old). Tumor volumes were monitored by calipers. Mice were injected with either PFCE (*N* = 3) or PFTBC (*N* = 3) via tail vein at a dose of 200 µL NE (PFCE: 285 mg/ml, PFTBC: 314.8 mg/ml). ^19^F MRI was acquired one day post-injection. Optionally, tumor tissues were harvested for CFT or flow cytometry. All animals were euthanized via CO_2_ gas asphyxiation in a closed container at a flow rate of 30–70% chamber volume displacement/min, followed by cervical dislocation. Tumor sizes, measured ex vivo, ranged from 0.5 to 1.2 cm length (L) and 0.5 to 1.1 cm width (W), with volumes of 0.65 to 0.78 cm^3^ as calculated using (L×W^2^×*pi*)/6. All mouse experiments were performed in accordance with protocols approved by the University of California San Diego Institutional Animal Care and Use Committee (IACUC; authorization #S13238) and complied with the National Institutes of Health Guide for the Care and Use of Laboratory Animals. All procedures involving mice were designed to minimize animal suffering and to reduce the number of animals used. This study is reported in accordance with the ARRIVE guidelines for the reporting of animal research.

### MRI

Mice were imaged using a Bruker 11.7 T MRI system and a 38 mm birdcage coil dual-tuned for ^1^H/^19^F. The ^19^F RARE images were acquired using TR/TE = 1000/15 ms, RARE factor 8, 450 averages (30 min acquisition time), and using 2 mm-thick axial slices, 35 mm square field of view and a 32 × 32 matrix. A 20 kHz excitation bandwidth was used with ^19^F frequency centered at -60 ppm or -92.5 ppm for PFTBC and PFCE, respectively. A 5 mm diameter calibrated ^19^F reference tube was included in images for quantification. Anatomical ^1^H RARE images were acquired using TR/TE = 1250/13.25 ms, RARE factor 8, 6 averages and using 1 mm-thick axial slices with a field of view identical to the ^19^F image and a 192 × 192 matrix. Images were co-registered using VivoQuant 2020 (VQ, Invicro, Boston, MA) for colocalization of ^19^F signals with anatomical structures. Using the same software, for each mouse, the total tumor ^19^F spin count was measured for the entire tumor volume across multiple ^19^F slices containing signal (typically 1–3), aided by the external calibrated ^19^F reference and the ^1^H scan for anatomical reference.

### CFT

A 6 × 7 cm mold was assembled on a base, and pre-chilled on dry ice. Optimal cutting temperature (OCT) medium was poured into mold. Excised tumors were embedded into the OCT and frozen overnight at -20 °C. The block was allowed to equilibrate in the chamber of the CFT instrument for 2 h. The entire block was serially imaged in 50 μm sections using white-light and with a red filter (555 nm laser, 585/11 nm filter). Acquired images were 4096 × 3008 pixels, with pixel size 18.3 × 50 μm. Data was reconstructed using proprietary software (Emit imaging, Boston, MA) applying subsurface correction and normalization, and transferred to Vivoquant software (InviCRO, Boston, MA) for visualization and analysis. For additional details regarding CFT, see reference^36^.

### Tumor dissociation

A 0.5 g sample of each 4T1 tumor was dissociated utilizing the Miltenyi Tumor Dissociation kit for mouse (cat. 130-096-730) following the manufacturer’s instructions, in conjunction with the manufacture’s gentleMACS Octo Dissociator product equipped with heaters and running under the program 37C_m_TDK_1. The dissociated cells were counted using the automatic cell counter Revvity Cellometer Ascend and ViaStain AOPI Staining Solution (cat. C52-0106).

### Flow cytometry

Tumor dissociated cells were stained for flow cytometry in 96 well plates. The cells were stained for 10 min at room temperature in the dark using a fixable viability dye (eFluor780, 1:1000, cat. 65-0865-14, eBioscience) diluted in 1× Dulbecco’s phosphate-buffered saline (dPBS), in the presence of Fc block (cat. 553141, BD) and True Stain Monocyte Blocker (cat. 426102, BioLegend). Following a wash with 1× dPBS containing 2% fetal bovine serum (FBS), cells were incubated with primary antibodies (Table 1) diluted 1:50–1:100 in 1× dPBS containing 2% FBS for 20 min at room temperature in the dark. Cells were washed twice in 1× dPBS in 2% FBS and fixed overnight in 1% PFA (BP531-25, Fisher BioREgents). Cells were analyzed using a FACSymphony A1 cell analyzer flow cytometer (Becton Dickinson), and the data were processed in FlowJo analysis software (Treestar/Becton Dickinson).

**Table 1:** Flow cytometry antibodies and dilutions.

Antibody	Clone	Vendor	Fluorophore	Catalog number	Dilution
Rat anti-mouse CD45	30-F11	Invitrogen	AF700	56-0451-80	1:50
Rat anti-mouse CD19	6D5	BioLegend	BV510	115,545	1:100
Rat anti-mouse CD3	17A2	BioLegend	BV421	100,227	1:100
Rat anti-mouse Ly-6G	1A8	BD Pharmingen	PE-Cy7	560,601	1:100
Rat anti-mouse CD11b	M1/70	BioLegend	AF488	101,217	1:100
Rat anti-mouse Ly-6 C	AL-21	BD Horizon	RB705	570,264	1:100
Rat anti-mouse F4/80	A3-1	Bio-Rad	BV650	MCA497SBV670	1:100

## Data Availability

Data available upon reasonable request.
